# Implementation and evaluation of a self-directed learning activity for first-year medical students

**DOI:** 10.1080/10872981.2020.1717780

**Published:** 2020-02-03

**Authors:** Molly Hill, Megan Peters, Michelle Salvaggio, Jay Vinnedge, Alix Darden

**Affiliations:** a Department of Microbiology and Immunology, University of Oklahoma Health Sciences Center, Oklahoma City, OK, USA; b Department of Pediatrics, University of Oklahoma Health Sciences Center, Oklahoma City, OK, USA; c Department of Medicine, University of Oklahoma Health Sciences Center, Oklahoma City, OK, USA; d St. Anthony Hospital Family Medicine Residency Program, PGY1, Family Medicine, St. Anthony’s Hospital, Oklahoma City, OK, USA

**Keywords:** Self-directed learning, preclinical curriculum, medical students, infectious disease case studies, self-reflection

## Abstract

**Background**: It is critical that medical students develop self-directed, life-long learning skills to navigate medical school successfully and to become competent healthcare professionals. Moreover, the Liaison Committee on Medical Education (LCME), the USA medical school accrediting body, requires activities designed to help students develop self-directed learning (SDL) skills in the preclinical years.

**Objective**: We evaluated the feasibility and effectiveness of a self-directed learning activity in a 6-week first-year medical student course.

**Design**: The course director assigned infectious disease case studies to teams of first-year medical students who individually assessed their knowledge gaps of the case, identified scholarly sources to fill their knowledge gaps, shared the information with their teammates, and reflected on their ability to guide their own learning. Students were asked to rate workload, team effort, acquisition of new clinical knowledge, and life-long learning skills. Students were also asked to reflect on how this assignment affected their perception of their SDL skills. Descriptive statistics were used to analyze responses to the Likert scale questions. Thematic analysis was applied to the comments.

**Results**: Survey response rate was 80% (131/163). Students strongly or moderately agreed that 1) they spent an appropriate amount of time on the project (94%), 2) the workload was evenly distributed among their teammates (95%), 3) their teammates made significant and timely contributions to the project (97%), 4) the project contributed to learning new clinical knowledge (92%), and 5) the project contributed to the acquisition of life-long learning skills (85%). The analysis team identified four themes from student reflections on their perception of their self-directed learning skills: self-learning skills, collaboration, application, and meta-cognition,

**Conclusions**: Study results demonstrated that we successfully implemented a case-based SDL activity in a first-year medical school course and that students perceived the activity as a valuable learning experience.

## Introduction

‘Everyone in healthcare has two jobs when they come to work every day: to do their work and to improve it.’[1] In the near future, medical knowledge will double every few months; therefore, it is critical that medical students develop life-long learning skills [2]. Self-directed learning (SDL) is considered an important component of life-long learning and thus is a key competency in medical school curricula [3]. Historically, self-directed learning was defined by Knowles as a process in which a learner takes the initiative, diagnoses their learning needs, creates learning goals, identifies resources for learning, applies appropriate learning strategies and evaluates their learning outcomes [4]. The Liaison Committee on Medical Education (LCME) [5] now requires medical schools to provide opportunities for students to participate in SDL activities as described in Standard 6.3 ‘The faculty of a medical school ensure that the medical curriculum includes self-directed learning experiences and time for independent study to allow medical students to develop the skills of lifelong learning. Skills that embody a lifelong learner include the ability to recognize one’s knowledge gaps, to know where and how to find credible sources with the relevant information, and then to synthesize this information and apply it to one’s clinical practice.’[5]

To develop self-directed learners, Knowles states that teachers must facilitate the acquisition of SDL skills [4]. Moreover, medical educators should not assume that medical students possess all the skills needed to engage successfully in SDL activities without training [6].

Implementing an SDL activity in a preclinical course at our institution presented several challenges including allotting time in a short, fast-paced course that is primarily lecture-based, providing individual feedback to a large class, designing SDL activities relevant to the course content, and assigning significant weight to the SDL grade. Therefore, the two goals of this study were to (1) determine the feasibility of including an SDL activity in a lecture-based preclinical medical school course and, (2) gain insight into the medical student perspective of engaging in the SDL activity.

## Materials and methods

### Assignment

This study was conducted according to the ethics guidelines of the University of Oklahoma Health Sciences Center. The Institutional Review Board granted an exemption for this project (IRB #7473). All first-year medical students (*N = 165*) were required to complete the SDL assignment but only those who signed individual consent forms (*N = 131)* were included in this study. The course director (MH) assigned a team-based SDL activity in a six-week course in microbiology and immunology taught during the spring semester of the first year. The assignment was designed in collaboration with a clinician specializing in infectious diseases (MS) and a fourth-year medical student (JV). When students matriculated, the Office of Medical Education assigned them to a team-based learning (TBL) group consisting of four to five students per group and they remained in these groups throughout the preclinical curriculum. Each TBL group was assigned an infectious disease case study (Appendix 1) taken from Partners in Infectious Diseases (https://www.idimages.org/idreview/). The cases corresponded to infectious agents covered during the course. The differential and final diagnoses for each case were blinded as well as images or content that would reveal the causative agent. Each student was required to identify one knowledge gap from the case study, fill in the knowledge gap with information from credible sources, and cite the references. Students were required to cite at least three references for the annotation of their knowledge gap and all citations had to include the PubMed reference number (PMID). The differential and final diagnoses developed by every team had to be supported with rationales and citations (three per infectious agent). The final product consisted of a team-based report with peer-reviewed publications to support their findings. The course director posted all materials related to this assignment to a learning management system (D2L). The assignment was explained during the course orientation but no further class time was spent demonstrating how to find and evaluate scholarly sources. However, the students were encouraged to consult the on-campus medical librarians for assistance with development of their search skills. The course director allotted an average of three hours of independent study time per week during the course to work on the assignment.

Student grades (2.5% of the final course grade) were determined using a rubric developed by the course director. The rubric was based on the learning outcomes of the assignment [7] which included the quality of the explanations of knowledge gaps, appropriateness of the scholarly sources, and format (Appendix 2). Scholarly sources were defined as journal articles with a PubMed Identifier (PMID) number. The course director did not grade the students on the accuracy of their differential and final diagnoses but rather on their ability to provide the rationale and supporting documentation to justify their conclusions. To assess the feasibility and impact of the SDL project, students completed a survey with both multiple choice and open-ended questions (Appendix 3). As part of the final assignment, each team was asked if they consulted a medical librarian.

### Data analysis

#### Quantitative analysis

A descriptive analysis using the survey responses obtained from the students to the five Likert-type questions analyzed. Percentages of responses were calculated for each question by dividing by the number of responses for each rating (Strongly Agree, Agree, Neither Agree nor Disagree, Disagree, and Strongly Disagree) by the total number of respondents and multiplying by 100% to determine the percentage per response.

#### Thematic analysis

Analysis of responses to the prompt, *Please reflect on how this assignment affected your perception of your self-directed learning skills*, was conducted using thematic analysis of open-ended questions [8]. A three-person analysis team that included two members with prior experience in qualitative research methods performed the analysis. The analysis team met eight times over three months to compare and reach consensus over code, category and theme development. The analysis process involved reading all written responses to the open-ended question and highlighting and naming the main ideas in each phrase. The highlighted words were then reviewed by the team to develop primary codes. After iterative independent coding, the team members met to compare their findings to assure consistency across all coded phrases and to eliminate codes not strongly iterated across the data. Primary codes were refined into a set of final codes by merging any primary codes that conveyed similar concepts into one final code. The analysis team identified commonalities across final codes and grouped them into broad categories. By cycling back through the data using constant comparison the analysis team developed the categories into named themes that captured the essence conveyed by the statements within each category. Significant statements that conveyed the overall sense of each of the themes were selected for presentation in this manuscript. The codes generated and associated statements were reviewed by all authors to ensure credibility of the analysis process.

## Results

### Student perspective on feasibility

Feasibility of the learning activity was enhanced by use of the team-based method because it provided an opportunity for the students to investigate a complex case. All members of a team were required to contribute to the differential and final diagnosis.

**Figure 1.**
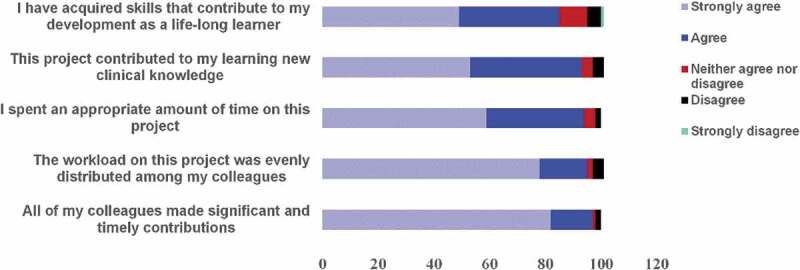
Student survey responses (n = 131) to likert-style questions following completion of SDL assignment

Students provided feedback regarding the time commitment and team-based approach. The majority of students (80%) reported that it took less than ten hours to complete the assignment and 94% of the students strongly or moderately agreed that their effort on this project was appropriate (Figure 1). The students strongly or moderately agreed that their teammates made significant and timely contributions (94%) and performed their share of the work (92%) (Figure 1). In addition, 15% of the teams reported that they consulted a medical librarian.

### Faculty perspective on feasibility

To make this project feasible, the course director used case studies (Partners Infectious Diseases) that correlated with course content. The detailed cases provided opportunity for each member of the team to identify knowledge gaps and search the literature to find clinically relevant answers. Using a three-item rubric, it was feasible for the course director to evaluate the assignment by the deadline for submitting grades. In order to reduce intrarater reliability [9], the course director made two passes through the assignments to review consistency in feedback provided to the students as well as point deductions. The course director assessed students on their ability to address their knowledge gaps with appropriate references in the correct format.

### Student perspective on engaging in an SDL activity

Student perspective of the SDL activity was assessed by asking them to respond to two survey questions and one open-ended question on what they learned by completing the assignment. The majority of students strongly or moderately agreed that they acquired life-long learning skills (85%) and new clinical knowledge (93%) (Figure 1).

Thematic analysis of the open-ended question, *Please reflect on how this assignment affected your perception of your self-directed learning skills*, resulted in the identification of seventeen primary codes. Three of these codes were eliminated after review and discussion by the analysis team due to redundancy or weak iteration across the data. After identifying connections and commonalities, the fourteen final codes were grouped into four themes, each containing three to four codes. For example, the codes *confidence, self-assessment, enjoyment, and challenged/stretched*, were grouped together in a theme, *Metacognition*, that related to internal, reflective processes. Table 1 provides details of the progression from final codes with sample statements to the themes. The four themes, *Self-learning skills, Collaboration, Application, and Metacognition*, represented the overarching concepts revealed through students’ responses to the open-ended question.

**Table 1.** Final codes with sample statements, themes and theme definitions

Final Codes	Sample Statements	Themes	Theme Definitions
Efficiency	I learned to be more efficient in identifying relevant sources that would answer the knowledge gaps that I had.	Self-learning Skills	Development of skills that facilitate acquisition of evidence-based knowledge, including use of library resources, time management strategies, and efficient ways of accessing and sorting through information. These skills were developed through practice.
Time management	Time management is something that I have had to relearn since starting medical school and I believe this self-directed learning assignment allowed me to reflect on my improved skills.		
Skill development	This assignment helped me learn how to streamline my search strategies to quickly find relevant peer reviewed journal articles.		
Practice	It also helped me to practice some of the evidence-based research evaluation techniques we learned in our PPS curriculum.		
Teamwork	I rarely get the opportunity to work in teams, so this project helped me build on my team work and leadership capabilities.	Collaboration	Development of teaming skills including communication, leadership, and utilization of available expertise to accomplish a common goal.
Learning from peers	I became aware how my research abilities were limited and asked peers how they researched to better improve my search.		
Expert consult	At the beginning of this assignment, I felt comfortable utilizing research databases. I soon realized that I was unfamiliar with a multitude of available resources, so I consulted a medical librarian.		
Professional identity	Self-directed learning is a method of continued learning that I will embrace as a practicing physician.	Application	Recognizing that the skills they’ve developed will transfer to their clinical practice.
Knowledge Application	It also reinforced my ability to synthesis basic science research and apply it to an understanding of aspects of disease processes.		
Experiential learning	Learning in lecture is just absorbing the material others have prepared for us, but with this project, not only did we have to interpret the clinical scenario presented, we had to apply that to searching the current scientific evidence.		
Confidence	This assignment made me more comfortable with a subject that I had very little confidence in. I have never taken Microbiology or Immunology before this course and having the chance to work on something that involved applying critical thinking skills and familiarizing myself with the literature in the subject was a nice break from the mundane multiple choice exams. If the course was only multiple choice exams, I don’t think I would have ever had the chance to grow confidence with this material.	Meta-cognition	Self-reflection on their interaction with the task that led to a new understanding of their weaknesses and abilities with respect to their research skills, addressed their confidence, and recognized their personal learning preferences.
Enjoyment	I enjoyed the SDL project because (it allowed me to identify my own weaknesses and address them via research. The personalization aspect was great because) I didn’t have to spend any time on things that I already knew, rather I got to focus on my own learning gaps and discuss them with a group.		
Self-assessment	I thought I was much better at doing research than I actually was and using this assignment to practice was very helpful.		
Challenged/stretched	This assignment pushed me to learn new and more efficient ways to access information to possibly help a patient.		

#### Self-learning skills

Students described self-learning skills that they perceived as being affected by the self-directed learning assignment. They commented on their improved efficiency searching databases and refined time-management skills as they participated in the assignment. ‘I learned to be more efficient in identifying relevant sources that would answer the knowledge gaps that I had.’ They perceived the SDL assignment as helping them refine existing skills and build new skills. ‘This assignment presented unique problems and forced me to grow as a researcher in order to adequately answer the questions presented to me.’

#### Collaboration

Students described the benefits and challenges of working as a team for the assignment. Several students talked about learning from their peers. ‘It not only taught me skills for self-learning and proper researching skills, but it allowed me to work from a team-based approach.’ Students also identified external partners, or expert consultants, who assisted their learning. ‘I have always struggled with researching articles and finding appropriate sources. For this assignment, I met with a librarian, and she was very helpful in giving me additional techniques for searching for scholarly articles.’

#### Application

The students frequently commented on the potential applications of the self-directed learning process to their future roles as clinicians. They identified similarities between the assignment and the expectations they had for themselves as professionals. ‘This assignment made me feel like I was on a real patient case.’ Additionally, students described positive impacts of the experiential learning process provided through the SDL assignment. ‘This assignment showed me that in reality medicine is not entirely black and white like it is on exam questions. The SDL provided great practice of integrating critical thinking, self-directed learning skills, and thinking outside the box. It helped me think about cases in a different way, considering multiple angles rather than just a sole cause (like an exam question).’

#### Metacognition

As the students reflected on their ability to guide their own learning, they made statements that conveyed an increased awareness of their individual strengths and knowledge gaps. ‘Prior to this assignment, I had felt that finding answers to specific questions was often difficult while using peer-reviewed sources. I came to realize that this was not the case and discovered the large number of specialty-centered journals there are available for use.’ Several students described feeling challenged by the assignment but they also expressed enjoyment of the learning activity. Some students reported increased confidence in their ability to perform self-directed learning, ‘This assignment made me feel more confident in my ability to research topics in the medical field and discern between relevant and irrelevant information.’ Others noted that they came into the task overly confident, ‘I thought I was much better at doing research than I actually was.’

Overall, student responses indicated that they became more self-aware of how they approached the process of learning. They gained tools that they will use for ongoing learning, including self-learning skills and effective use of collaborators. In addition, they identified how self-directed learning applies to their future roles as clinicians.

## Discussion

The primary educational goal of this assignment was to provide an opportunity for students to develop and practice skills essential for life-long learning. The course director implemented a team-based SDL activity for first-year medical students that met the criteria outlined by LCME for SDL [5]. Many medical schools address these criteria by integrating the principles of SDL into problem-based [10], team-based [11], or case-based [12] learning curricula. In a preclinical curriculum that is primarily lecture-based, course activities must be intentionally designed to promote SDL skills [13].

Our first research question was to determine the feasibility of including an SDL activity in a lecture-based preclinical medical school course. This study served to confirm that our method of implementing an SDL activity in a preclinical course was achievable from the perspective of both the students and the course director. Similar to our team-based approach, a number of studies report using clinical cases in lecture-based courses to promote self-directed learning [14–16]. However, these studies differ from the design of our SDL activity in several ways. First, our assignment did not replace lecture material. Secondly, we did not provide facilitators nor any additional resources such as assigned readings. Finally, we assessed course products for use of scholarly sources and synthesis of information. Numerous assessments have been published that evaluate skills in information literacy and evidence-based medicine [17,18]; however these are beyond the scope of this assignment or require time and resources not available in this study. Turnbow and Evener [19] reported using a modified version of the Valid Assessment of Learning in Undergraduate Education (VALUE) rubric to assess information literacy skills of graduate health sciences students. Utilization of a similar rubric minimized the grading burden on the instructor while still providing feedback to the students, an essential component of SDL [4,17,18,20]. Our study adds to the literature focusing on pre-clinical medical student courses specifically by describing an SDL activity that can be implemented in lecture-based courses with minimal faculty involvement.

Our SDL assignment was designed to promote acquisition of search skills needed to address current and future knowledge gaps. In other studies, scholars have compared knowledge acquisition by students using an SDL approach versus other more traditional modes of content delivery [21–24]. Murad et al. conducted a systematic review of the effectiveness of SDL in the health professions [25]. They found moderate quality evidence that SDL activities result in gains in the knowledge domain when compared to traditional teaching methods. In our study, however, we focused more on our second research question, which investigated how the assignment changed their perception of their SDL skills rather than acquisition of case-specific knowledge.

The theme *self-learning skills* contained the greatest volume of student comments, many of which referred to time management, skill development, improved efficiency, and practice. Deficiencies in time management, among others, is one of several ‘generic skills’ [26] identified by struggling medical students [27]. An important skill of the self-directed learner is the ability to identify and access scholarly resources [4]. The finding that a number of our preclinical students report having insufficient search skills is borne out by other studies. Richardson et al [28]. surveyed incoming medical students at Weill Cornell Medical College to determine their familiarity with information resources and services. Approximately half (53%) of the students stated having used an NCBI bioinformatics database; however, the majority of the students were interested in learning more about how to use the tools. In another study, first-year dental students were assessed on their information literacy skills. When asked to provide evidence-based citations to support their responses to the questions in the assessment, one-third to one-half of students could not find appropriate citations [29]. A review by Just (2012) [30] concluded that there is little evidence that information literacy skills persist following training. Whether or not our students’ perceived improvement in search skills will persist throughout their training is a future research question.

Another theme we identified from students’ reflections was *collaboration*. Placing students in teams added an opportunity for peer support. Students reported collaborating with peers and medical librarians for assistance with searching the literature. Many medical schools integrate information literacy into the preclinical curriculum by utilizing medical librarians to provide instruction in information seeking skills and/or embedding them as instructors and participants in the curriculum [31–33]. At Albert Einstein College of Medicine, librarians developed tutorials for first- and second-year medical students on conducting literature searches. The tutorials comprised the out-of-class component of a flipped classroom approach [33] and were well received by the students. Without a course per se on information literacy, SDL activities become essential to promote development of the skills students need in order to find clinically relevant information.

The theme *application* captured the perception of students that they were learning skills essential to their future role as clinicians. Students must be aware of the need to develop strategies for coping with the explosion of medical and scientific data [2,34]. Shojania et al. reported that high-quality systematic reviews covering randomized trials of conventional drugs, devices, or procedures had a median survival time of 5.5 years before they required an update [35]. Effective SDL activities should alert students to the challenges they will face in curating the information they need to make clinical decisions.

Another theme we identified, *metacognition*, the ability to think about one’s thinking [36], is an essential skill for self-directed learning. Some medical educators are calling for medical schools to teach metacognitive skills [37–39]. In fact, reflection, or continual self-assessment, is one of the frequently listed strategies for teaching metacognitive skills in the health professions [37,40,41]. Reflection is critical in the training of future physicians as they develop clinical reasoning skills and provide evidence-based care. The inability to reflect on one’s own clinical practice often results in diagnostic and treatment errors [37]. In this study, we identified student reflections that relate to the three components of metacognition: planning, monitoring, and evaluating [40]. Asking students to reflect on their perception of their SDL skills was a valuable component of this activity because students were able to identify their own strengths and weaknesses. This activity has made us aware of the need to incorporate more opportunities for self-reflection in our preclinical coursework.

The theoretical literature of SDL in adults [4,6,42] and the evolving literature on SDL in medical education [43–45] describe multiple dimensions of SDL including (1) management of learning skills, (2) personality characteristics of the learner, (3) the learning environment, and (4) the metacognitive process [6,46,47]. The results of our study build on this literature by showing that our students’ perceptions of how they approach SDL mirror these dimensions.

One strength of this study was the student response rate for the open-ended question (80%). A weakness of this study is that it was conducted it at one institution and in one first-year medical student course and may not generalize to other courses and settings. We recommend evaluation of the readiness of incoming medical students for SDL activities [48] and implementation of activities that enhance their SDL skills. Overall, the student reflective comments provided insight into how this activity affected their SDL abilities beyond what we could observe by simply grading their assignments. The students’ reflections revealed that we should not assume all of our medical students matriculate with well-developed self-directed learning skills, an observation which is consistent with the SDL literature [4,6,20,25].

In summary, we recommend that SDL assignments occur regularly throughout the pre-clinical curriculum to promote life-long learning skills. This study demonstrates that SDL assignments can be successfully implemented in pre-clinical courses and are valued by the students as contributing to the skills they view as necessary for delivering effective patient care.

## Appendix 1. List of case studies from Partners in Infectious Diseases

Case #02048: A man in his fifties with rapidly progressive fever and leg swelling.

[Internet]. Partners Infectious Disease Images. Available from: http://www.idimages.org/idreview/case/caseid=331

Figure 3 courtesy of Lynn Bry, MD.

Copyright Partners Healthcare System, Inc. All rights reserved.

Case #03006: A man with fever, pulmonary infiltrates, abnormal liver function tests and anemia. [Internet]. Partners Infectious Disease Images. Available from: http://www.idimages.org/idreview/case/caseid=128

Copyright Partners Healthcare System, Inc. All rights reserved.

Case #15,007: 2015 IDWeek Case: A woman with AIDS, Kaposi sarcoma and acute respiratory distress. [Internet]. Partners Infectious Disease Images. Available from: http://www.idimages.org/idreview/case/caseid=513

Copyright Partners Healthcare System, Inc. All rights reserved.

Case #07009: A teenager with headache, vomiting and a new rash. [Internet]. Partners Infectious Disease Images. Available from: http://www.idimages.org/idreview/case/caseid=10

Copyright Partners Healthcare System, Inc. All rights reserved.

Case #08009: A woman in her thirties with fevers and a pruritic rash. [Internet]. Partners Infectious Disease Images. Available from: http://www.idimages.org/idreview/case/caseid=26.

Copyright Partners Healthcare System, Inc. All rights reserved.

Case #09010: A young woman with pre-auricular pain, erythema and swelling on the left side of the face. [Internet]. Partners Infectious Disease Images. Available from: http://www.idimages.org/idreview/case/caseid=56

Copyright Partners Healthcare System, Inc. All rights reserved.

Case #10,001: An elderly woman with right arm and shoulder pain. [Internet]. Partners Infectious Disease Images. Available from: http://www.idimages.org/idreview/case/caseid=66

Copyright Partners Healthcare System, Inc. All rights reserved.

Case #10,004: A woman with right upper quadrant pain, radiating to the right shoulder, and a cystic mass in the right upper quadrant. [Internet]. Partners Infectious Disease Images. Available from: http://www.idimages.org/idreview/case/caseid=69

Copyright Partners Healthcare System, Inc. All rights reserved.

Case #03015: A man with fever and wasting. [Internet]. Partners Infectious Disease Images. Available from: http://www.idimages.org/idreview/case/caseid=138

Copyright Partners Healthcare System, Inc. All rights reserved.

Case #10,014: A teen-aged girl presented with anemia, fever and a rash. [Internet]. Partners Infectious Disease Images. Available from: http://www.idimages.org/idreview/case/caseid=168

Copyright Partners Healthcare System, Inc. All rights reserved.

Case #11,009: 2011 IDSA Case: A man with fevers, vomiting, and cyanosis of the feet. [Internet]. Partners Infectious Disease Images. Available from: http://www.idimages.org/idreview/case/caseid=230

Copyright Partners Healthcare System, Inc. All rights reserved.

Case #08002: An elderly woman with pain in the right shoulder and skin changes of the left forearm five weeks after surgery on the left arm. [Internet]. Partners Infectious Disease Images. Available from: http://www.idimages.org/idreview/case/caseid=19

Copyright Partners Healthcare System, Inc. All rights reserved.

Case #16,002: A man in his twenties with recurrent fevers, foot swelling, dyspnea and bradycardia. [Internet]. Partners Infectious Disease Images. Available from: http://www.idimages.org/idreview/case/caseid=519

Copyright Partners Healthcare System, Inc. All rights reserved.

Case #16,009: A man with a rash and an axillary mass. [Internet]. Partners Infectious Disease Images. Available from: http://www.idimages.org/idreview/case/caseid=526

Copyright Partners Healthcare System, Inc. All rights reserved.

Case #10,003: A man in his seventies with fevers and confusion. [Internet]. Partners Infectious Disease Images. Available from: http://www.idimages.org/idreview/case/caseid=68

Copyright Partners Healthcare System, Inc. All rights reserved.

Case #14,009: 2014 IDWeek Case: A man with tuberculosis and new masses in the neck and the thigh. [Internet]. Partners Infectious Disease Images. Available from: http://www.idimages.org/idreview/case/caseid=504

Copyright Partners Healthcare System, Inc. All rights reserved.

Case #16,008: A young man with a diffuse rash after travel to Madagascar. [Internet]. Partners Infectious Disease Images. Available from: http://www.idimages.org/idreview/case/caseid=525. Figure 1, 2, and 7 were provided by Dr. Maroun Sfeir and Figures 3, 4, 5 and 6 were provided by Dr. Cynthia Magro.

Copyright Partners Healthcare System, Inc. All rights reserved.

Case #02035: A man with fever and a gangrenous leg. [Internet]. Partners Infectious Disease Images. Available from: http://www.idimages.org/idreview/case/caseid=318

Copyright Partners Healthcare System, Inc. All rights reserved.

Case #11,002: A pregnant woman with HIV infection and fever, decreased hearing and painful lesions in the left ear. [Internet]. Partners Infectious Disease Images. Available from: http://www.idimages.org/idreview/case/caseid=195

Copyright Partners Healthcare System, Inc. All rights reserved.

Case #12,001: A young woman with respiratory failure and supraventricular tachycardia. [Internet]. Partners Infectious Disease Images. Available from: http://www.idimages.org/idreview/case/caseid=445

Copyright Partners Healthcare System, Inc. All rights reserved.

Case #15,011: 2015 IDWeek Case: A woman with progressive skin rash and ulcers. [Internet]. Partners Infectious Disease Images. Available from: http://www.idimages.org/idreview/case/caseid=517

Copyright Partners Healthcare System, Inc. All rights reserved.

Case #06017: A man with HIV and AIDS and a skin rash. [Internet]. Partners Infectious Disease Images. Available from: http://www.idimages.org/idreview/case/caseid=111

Copyright Partners Healthcare System, Inc. All rights reserved.

Case #13,014: 2013 IDWeek Case: A man with shock, multiorgan failure and DIC after a one-week illness. [Internet]. Partners Infectious Disease Images. Available from: http://www.idimages.org/idreview/case/caseid=490

Copyright Partners Healthcare System, Inc. All rights reserved.

Case #02009: A woman with HIV infection and AIDS and profuse watery diarrhea. [Internet]. Partners Infectious Disease Images. Available from: http://www.idimages.org/idreview/case/caseid=183

Copyright Partners Healthcare System, Inc. All rights reserved.

Case #02010: A boy with a rash, gastrointestinal symptoms and a fever. [Internet]. Partners Infectious Disease Images. Available from: http://www.idimages.org/idreview/case/caseid=196

Copyright Partners Healthcare System, Inc. All rights reserved.

Case #08003: A woman with fevers and skin lesions after surgery. [Internet]. Partners Infectious Disease Images. Available from: http://www.idimages.org/idreview/case/caseid=20

Copyright Partners Healthcare System, Inc. All rights reserved.

## Appendix 2. Self-directed learning rubric

**Table:**

Element	Excellent (90–100)	Average (80–89)	Poor (<80)	Points
Annotations **30% of grade**	All annotations go beyond class material and are clinically relevant; include the latest advances, if applicable and clearly and succinctly described 27–30 points	Most annotations go beyond class material and are clinically relevant; include the latest advances, if applicable and adequately described 24–26.7 points	Annotations are regurgitation of class material, lack clinical application, or poorly described <24 points	
Scholarly sources **60% of grade**	All citations are scholarly and peer-reviewed; search strategy included’ PMID included on all citations 54–60 points	Most citations are scholarly and peer-reviewed; search strategy included; PMID included on most citations 48–53.4 points	Some citations are scholarly and peer-reviewed and/or no search strategy included; no PMID included on citations <48 points	
Format **10% of grade**	Essentially free of spelling and formatting errors; followed all instructions; everyone used the same font and citation style 9–10 points	Some errors in text and/or inconsistent formatting; followed most instructions; inconsistent use of fonts and citation style 8–8.9 points	Numerous errors and/or inconsistent formatting; failed to follow instructions; everyone used different fonts and citation fonts <8 points	
TOTAL				

## Appendix 3. Self-directed Learning (SDL) Survey

(Note: This survey administered on-line in D2L, a course management program.)

In order to complete the SDL learning exercise, you must complete this survey. The purpose of this survey is 1) to provide educational *quality improvement* data to ensure that the SDL assignment is a valuable learning experience. If you signed the consent form at the beginning of the course 2), these data will be included in a research study on the effectiveness of self-directed learning activities. Since SDL leaning activities are required by the accreditation body that governs medical schools, Liaison Committee on Medical Education (LCME), findings from this study will be shared with colleagues and contribute to the evidence-based medical education literature.

- R
- Agree
- Neither Agree nor Disagree
- Agree
- Strongly Agree

3. Please reflect on how this assignment affected your perception of your self-directed learning skills.

4. Please reflect on how your SDL skills will contribute to your development as a life-long learner.
